# Study Protocol: The Heart and Brain Study

**DOI:** 10.3389/fphys.2021.643725

**Published:** 2021-03-31

**Authors:** Sana Suri, Daniel Bulte, Scott T. Chiesa, Klaus P. Ebmeier, Peter Jezzard, Sebastian W. Rieger, Jemma E. Pitt, Ludovica Griffanti, Thomas W. Okell, Martin Craig, Michael A. Chappell, Nicholas P. Blockley, Mika Kivimäki, Archana Singh-Manoux, Ashraf W. Khir, Alun D. Hughes, John E. Deanfield, Daria E. A. Jensen, Sebastian F. Green, Veronika Sigutova, Michelle G. Jansen, Enikő Zsoldos, Clare E. Mackay

**Affiliations:** ^1^Department of Psychiatry, Warneford Hospital, University of Oxford, Oxford, United Kingdom; ^2^Oxford Centre for Human Brain Activity, Wellcome Centre for Integrative Neuroimaging, University of Oxford, Oxford, United Kingdom; ^3^Oxford Institute of Biomedical Engineering, University of Oxford, Oxford, United Kingdom; ^4^Institute of Cardiovascular Science, University College London, London, United Kingdom; ^5^FMRIB Centre, Wellcome Centre for Integrative Neuroimaging, University of Oxford, Oxford, United Kingdom; ^6^Nuffield Department of Clinical Neurosciences, University of Oxford, Oxford, United Kingdom; ^7^Radiological Sciences, Division of Clinical Neuroscience, School of Medicine, University of Nottingham, Nottingham, United Kingdom; ^8^Sir Peter Mansfield Imaging Centre, School of Medicine, University of Nottingham, Nottingham, United Kingdom; ^9^Nottingham Biomedical Research Centre, Queens Medical Centre, University of Nottingham, Nottingham, United Kingdom; ^10^School of Life Sciences, University of Nottingham, Nottingham, United Kingdom; ^11^Department of Epidemiology and Public Health, University College London, London, United Kingdom; ^12^Inserm U1153, Epidemiology of Ageing and Neurodegenerative Diseases, Paris, France; ^13^Mechanical Engineering, Brunel University London, Uxbridge, United Kingdom; ^14^MRC Unit for Lifelong Health and Ageing, Institute of Cardiovascular Science, University College London, London, United Kingdom; ^15^Department of Neurology, Donders Institute for Brain, Cognition and Behaviour, Radboud University Medical Center, Nijmegen, Netherlands

**Keywords:** ageing, MRI, cerebrovascular reactivity, cognition, dementia prevention, longitudinal cohort, ultrasound, heart-brain

## Abstract

**Background:**

It is well-established that what is good for the heart is good for the brain. Vascular factors such as hypertension, diabetes, and high cholesterol, and genetic factors such as the apolipoprotein E4 allele increase the risk of developing both cardiovascular disease and dementia. However, the mechanisms underlying the heart–brain association remain unclear. Recent evidence suggests that impairments in vascular phenotypes and cerebrovascular reactivity (CVR) may play an important role in cognitive decline. The *Heart and Brain Study* combines state-of-the-art vascular ultrasound, cerebrovascular magnetic resonance imaging (MRI) and cognitive testing in participants of the long-running Whitehall II Imaging cohort to examine these processes together. This paper describes the study protocol, data pre-processing and overarching objectives.

**Methods and Design:**

The 775 participants of the Whitehall II Imaging cohort, aged 65 years or older in 2019, have received clinical and vascular risk assessments at 5-year-intervals since 1985, as well as a 3T brain MRI scan and neuropsychological tests between 2012 and 2016 (Whitehall II Wave MRI-1). Approximately 25% of this cohort are selected for the *Heart and Brain Study*, which involves a single testing session at the University of Oxford (Wave MRI-2). Between 2019 and 2023, participants will undergo ultrasound scans of the ascending aorta and common carotid arteries, measures of central and peripheral blood pressure, and 3T MRI scans to measure CVR in response to 5% carbon dioxide in air, vessel-selective cerebral blood flow (CBF), and cerebrovascular lesions. The structural and diffusion MRI scans and neuropsychological battery conducted at Wave MRI-1 will also be repeated. Using this extensive life-course data, the *Heart and Brain Study* will examine how 30-year trajectories of vascular risk throughout midlife (40–70 years) affect vascular phenotypes, cerebrovascular health, longitudinal brain atrophy and cognitive decline at older ages.

**Discussion:**

The study will generate one of the most comprehensive datasets to examine the longitudinal determinants of the heart–brain association. It will evaluate novel physiological processes in order to describe the optimal window for managing vascular risk in order to delay cognitive decline. Ultimately, the *Heart and Brain Study* will inform strategies to identify at-risk individuals for targeted interventions to prevent or delay dementia.

## Introduction

The majority of dementia patients have cardiovascular co-morbidities, and dementia and cardiovascular diseases share many risk factors (Poblador-Plou et al., 2014). These include vascular factors such as high blood pressure, high cholesterol, smoking, diabetes, and physical inactivity, as well as genetic factors, such as carrying the APOE4 allele (Qiu and Fratiglioni, 2015). Vascular risk is potentially treatable, and managing it alongside other modifiable lifestyle factors could prevent an estimated 40% of dementia cases (Livingston et al., 2020). Prevention on this scale would substantially improve quality of life for a society which in the next 50 years is predicted to see a near tripling in the prevalence of dementia (Patterson, 2018). However, maximizing such dementia prevention efforts requires an understanding of (1) *how* and (2) *when in the lifespan* vascular risk factors affect brain and cognitive health, and (3) *who* is most likely to benefit from preventative interventions. The *Heart and Brain Study* combines detailed vascular phenotyping and state-of-the-art magnetic resonance imaging (MRI) of the brain in a longitudinal prospective ageing cohort to investigate these questions with three overarching objectives.

The first objective is to examine the emerging role of cerebrovascular regulation in the pathway from vascular risk to cognitive decline. Impairments in cerebrovascular reactivity (CVR) have a well-established role in vascular dementia but growing evidence suggests that CVR can contribute to cognitive decline and Alzheimer’s disease (Cantin et al., 2011; Yezhuvath et al., 2012). CVR is the ability of the cerebral blood vessels to modulate their vasomotor tone to optimize cerebral perfusion in response to a physiological challenge. This process is essential for maintaining stable brain oxygenation and is a marker of the brain’s vascular reserve; higher CVR reflects better cerebrovascular health (Glodzik et al., 2013). Impairments in CVR can, over time, result in cerebral hyper- or hypoperfusion, with the latter being a well-established mechanism in the dementia pathway, occurring years before the appearance of clinical symptoms (Iturria-Medina et al., 2016). Studies have reported reduced CVR in mouse models of Alzheimer’s disease (Nicolakakis et al., 2008), in Alzheimer’s and vascular dementia (Coleman and Flood, 1987; Cantin et al., 2011; Yezhuvath et al., 2012; Gao et al., 2013; Alwatban et al., 2019), and individuals with established dementia co-morbidities such as stroke, hypertension, and diabetes (Troisi et al., 1998; Petrica et al., 2007; Thrippleton et al., 2017). CVR therefore stands to be a powerful early biomarker of dementia, even preceding established MRI biomarkers such as hippocampal atrophy. However, there are still substantial limitations in our understanding of this process. Most CVR imaging studies have utilized either positron emission tomography, which is expensive and exposes participants to ionizing radiation, or transcranial doppler which lacks spatial resolution and limits an understanding of regional variations in CVR (Silvestrini et al., 2006; Vicenzini et al., 2007; Gao et al., 2013; Catchlove et al., 2018). In addition, studies vary in the choice of vasodilators used to elicit the CVR response; with recent reviews suggesting that stimuli such as acetazolamide, breath-holding, and re-breathing may not be as robust or reproducible as the use of carbon dioxide (CO_2_)-enriched air (Fierstra et al., 2013). There have also been calls to assess the tolerability of and compliance with hypercapnia fMRI challenges in elderly and at-risk populations (Moreton et al., 2016). Further, while there is promising evidence supporting a role for CVR in cognitive impairment, we currently lack an understanding of the relationship between CVR and (a) circulatory disturbances in the blood vessels supplying the brain such as the aorta and carotid arteries, (b) other cerebrovascular anomalies such as changes in cerebral blood flow (CBF) and vascular lesions, and (c) longitudinal brain morphology and microstructure. Examining these processes together would provide insights into how alterations in system haemodynamics can affect the brain’s delicate microvasculature. The *Heart and Brain Study* uses the latest advances in non-invasive and spatially sensitive blood oxygen level dependent (BOLD) fMRI to measure CVR in response to a 5% CO_2_ stimulus in order to overcome these methodological limitations and address the gaps in our current understanding.

The second objective of this study is to further understand the temporal dynamics of dementia risk factors. Risk factors have been shown to have different effects across the life course. For instance, while mid-life cholesterol, blood pressure and obesity are associated with higher dementia incidence, later-life measures of these risk factors are associated with a lower incidence of dementia (Martín-Ponce et al., 2010). This is interesting as it suggests that risk factors may also have age-specific effects on the underlying cerebrovascular anomalies, possibly depending on whether the factors have been assessed before or during the preclinical stage of dementia. Indeed, we have previously shown age effects of vascular risk factors on grey matter (GM) volume, white matter (WM) microstructure (Zsoldos et al., 2020), cerebral perfusion (Suri et al., 2019) and clinical dementia (Singh-Manoux et al., 2017). However, there is an evident lack of longitudinal neuroimaging studies which assess the age-specific effects of dementia risk on CVR and other measures of cerebrovascular ageing. The *Heart and Brain Study* will investigate these age-specific effects by recruiting participants from the longitudinal Whitehall II Imaging Study, who have already received detailed clinical follow-ups every 5 years starting from mean age 47.8 years (at Wave 3), as well as a multi-modal brain MRI scan and neuropsychological tests at mean age 70 years (2012–2016) (Marmot and Brunner, 2005; Filippini et al., 2014). By capitalizing on this uniquely comprehensive dataset, the study will examine the association of 30-year antecedent trajectories of vascular risk with cerebrovascular regulation and cognitive decline in older age.

Third, on the question of personalised interventions, it has been shown that lifestyle modifications may be more beneficial for APOE4-carriers than non-carriers, however, the reasons for this remain unclear (Brown et al., 2012; Gardener et al., 2015). Interestingly, both younger (Suri et al., 2014a) and older (Hajjar et al., 2015; Wolters et al., 2016). APOE4 carriers have been shown to have lower CVR than non-carriers. This is important because impairments in CVR are likely reversible through exercise interventions (Ivey et al., 2011; Murrell et al., 2013), statins (Forteza et al., 2012), and acetylcholinesterase inhibitors such as galantamine and donepezil (Rosengarten et al., 2006; Bar et al., 2007), the latter being established treatments for Alzheimer’s disease. This evidence (albeit preliminary) positions CVR as a promising early marker for identifying at-risk individuals eligible for targeted interventions, and as a relevant surrogate endpoint in clinical trials. This study will therefore examine whether associations between CVR, vascular risk and cognitive impairment are moderated by the presence of the APOE4 risk allele.

In this paper, we describe the design, organisation, and participant inclusion criteria for the *Heart and Brain Study*. We present the protocols for acquisition and pre-processing of vascular measurements, MRI scans, and cognitive and mental health assessments. We discuss preliminary findings on the feasibility of hypercapnia fMRI and describe the key hypotheses for the study.

## Methods and Analysis

### Study Design

**FIGURE 1:**
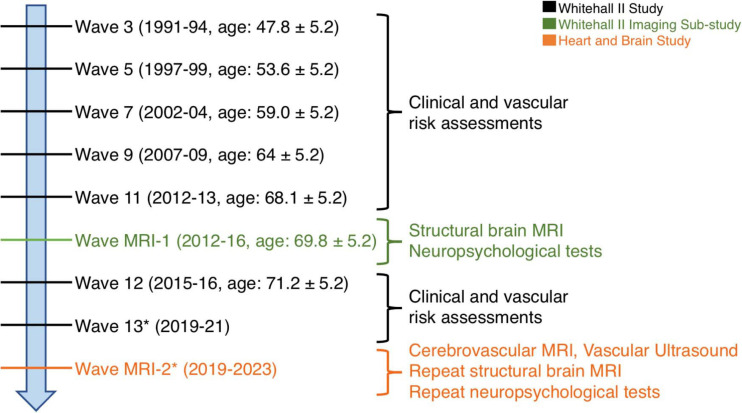
Study and cohort design. Clinical and vascular risk information was collected at the seven out of 13 waves of the Whitehall II Study, marked in black. APOE genotyping was performed at Wave 7. Brain MRI and cognitive assessments were collected at Wave MRI-1 (Whitehall II Imaging Sub-study), marked in green. A sub-set of the MRI and cognitive protocol are repeated at Wave MRI-2 (*Heart and Brain Study*), with novel cerebrovascular MRI and vascular ultrasound scans, marked in orange. *Indicates waves that are in progress at the time of publication.

Participants of the *Heart and Brain Study* are selected from the Whitehall II Imaging Sub-study cohort (Filippini et al., 2014). This cohort comprises 775 retired British civil servants who received multi-modal brain structural and functional MRI scans as well as a detailed battery of cognitive tests and mental health assessments between April 2012 and December 2016 at the Centre for Functional Magnetic Resonance Imaging of the Brain (FMRIB), now part of the Wellcome Centre for Integrative Neuroimaging (WIN), University of Oxford. Cohort participants have also undergone repeated longitudinal clinical and cognitive testing since 1985, across 13 Waves of the parent Whitehall II Study at University College London (Marmot and Brunner, 2005). A description of the longitudinal study design and measures collected at the Waves which included clinical follow-ups are provided in Figure 1. The *Heart and Brain Study* will recruit 200 participants from the Wave MRI-1 cohort for a follow-up visit at the Oxford Centre for Human Brain Activity (OHBA) within the WIN. For consistency, the Whitehall II Imaging Sub-study and the *Heart and Brain Study* waves will be referred to as “Wave MRI-1 (2012–2016)” and “Wave MRI-2 (2019–2023),” respectively.

The testing protocol for the *Heart and Brain Study* consists of a single 3-h session comprising a 45-min MRI scan, 1 h of physiological and vascular measurements, 1 h of cognitive tests, and a self-administered health questionnaire that is completed at home in the week prior to the testing appointment (Table 1).

**TABLE 1:** Assessments for the Whitehall II Imaging Sub-study (Wave MRI-1) and *Heart and Brain Study* (Wave MRI-2).

	Wave MRI-1	Wave MRI-2

Study name	Whitehall II Imaging Sub-study	Heart and Brain Study
Duration	2012–2016	2019–2023
**Cognitive tests**		
Montreal Cognitive Assessment	X	X
Trail Making Test, A and B	X	X
Rey Complex Figure Test	X	X
Categorical Fluency Test (animals)	X	X
Hopkins Verbal Learning Test-R	X	X
Boston Naming Test-60	X	X
Digit Span	X	X
Digit Coding Tests	X	X
Test of Premorbid Functioning	X	X
Dots and Letters	X	
Executive Clock Drawing Task (CLOX)	X	
CANTAB Reaction Time	X	
Purdue Pegboard	X	
**Physiological measurements**		
Aortic ultrasound		X
Carotid ultrasound		X
Central blood pressure		X
Body mass index	X	X
Peripheral blood pressure	X	X
**MRI (core sequences)**		
T1-weighted	X	X
Diffusion-weighted	X	X
Fluid attenuated inversion recovery (FLAIR)	X	X
Fieldmaps	X	X
T2*-weighted	X	
Resting-state MB6 BOLD	X	
Respiratory-calibrated MB6 BOLD		X
Vessel-encoded pCASL		X
T2-relaxation-under-spin-tagging (TRUST)		X
**Clinical questionnaires (self-administered)**		
General Health Questionnaire	X	X
Mood Disorder Questionnaire	X	X
Centre for Epidemiological Studies Depression Scale	X	X
State and Trait Anxiety Inventory	X	X
Physical Activity Questionnaire for Older Adults	X	X
Locus for Causality Exercise Questionnaire	X	X
Pittsburgh Sleep Quality Index	X	X
Jenkins Sleep Questionnaire	X	X
Life-Orientation Revised	X	X
Life Events	X	X
MacArthur stress reactivity questionnaire	X	X
Penn State Worry Questionnaire	X	X
Handedness	X	X
Medical history (hospitalisations, diagnoses, and medications)	X	X
Alcohol and smoking	X	X
5-Dimensional Curiosity Scale		X
Demographics (age, sex, and education)	X	X

*PCASL: pseudocontinuous arterial spin labelling; BOLD: blood oxygen level dependent, MB6: multiband acceleration factor 6.*

Due to an MRI scanner upgrade two-thirds of the way through Wave MRI-1, two scanners were used for the baseline scans: a 3T Siemens Magnetom Verio scanner with a 32-channel head coil (*n* = 552, April 2012–December 2014) and a 3T Siemens Prisma Scanner with a 64-channel head-neck coil, both at the FMRIB Centre, Oxford (*n* = 223, July 2015–December 2016) (Filippini et al., 2014). *Wave MRI-2* is conducted on a 3T Prisma scanner at the OHBA Centre, Oxford. All core imaging sequences have been matched as closely as possible between scanners and are described in Table 2.

**TABLE 2:** Acquisition parameters of the core MRI sequences at Waves MRI-1 and MRI-2.

MRI	Wave MRI-1 3T Verio	Wave MRI-1 3T Prisma	Wave MRI-2 3T Prisma
Study name	Whitehall II Imaging	Whitehall II Imaging	*Heart and Brain Study*
Duration	April 2012–December 2014	July 2015–December 2016	October 2019–2023
*N*	550	225	200
Head coil	32-channel	64-channel	64-channel
**Structural T1**	MEMPR	MPRAGE	MPRAGE
Voxel, mm^3^	1 × 1 × 1	1 × 1 × 1	1 × 1 × 1
TR, ms	2,530	1,900	1,900
TE, ms	1.79/3.65/5.51/7.37	3.97	3.97
TI, ms	1,380	904	904
Flip angle,°	7	8	8
Field of view, mm	256	192	192
Acquisition time	6 min 12 s	5 min 31 s	5 min 31 s
**Diffusion MRI**			
Voxel, mm^3^	2 × 2 × 2	2 × 2 × 2	2 × 2 × 2
TR/TE, ms	8,900/91.2	8,900/91	8,900/91
b-value, s/mm^2^	1,500	1,500	1,500
*N* volumes (A >> P)	60 + 5*b* = 0 s	60 + 5*b* = 0 s	60 + 5*b* = 0 s
*N* volumes (P >> A)	1*b* = 0	1*b* = 0	1*b* = 0
Field of view, mm	192	192	192
Acquisition time	9 min 56 s	10 min 5 s	10 min 5 s
**FLAIR**			
Voxel, mm^3^	0.4 × 0.4 × 3.0	0.4 × 0.4 × 3.0	0.4 × 0.4 × 3.0
TR/TE, ms	9,000/73	9,000/73	9,000/73
TI, ms	2,500	2,500	2,500
Flip angle, °	150	150	150
Field of view, mm	220	220	220
Acquisition time	4 min 14 s	4 min 14 s	4 min 14 s
**Fieldmaps**			
Voxel, mm^3^	3 × 3 × 3	3 × 3 × 3	3 × 3 × 3
TR, ms	400	378	590
TE, ms	5.19/7.65	4.92/7.38	4.92/7.38
Flip angle, °	60	45	46
Field of view, mm	258	192	216
Acquisition time	1 min 11 s	49 s	1 min 26 s
**Respiratory-calibrated BOLD functional MRI**			
Voxel, mm^3^	–	–	2.4 × 2.4 × 2.4
TR/TE, ms	–	–	800/30
Flip angle, °	–	–	52
Field of view, mm	–	–	216
Multi-band acceleration factor	–	–	6
*N* volumes	–	–	450
Acquisition time	–	–	6 min 8 s
Vessel-encoded pCASL*			
Voxel, mm^3^	–	–	3.4 × 3.4 × 4.5
TR/TE, ms	–	–	4,400 (variable)/14
Labelling duration, ms			1,400
Phase partial Fourier	–	–	6/8
Post-labelling delays, ms	–	–	250/500/750/1,000/1,250/1,500/1,750
Slices	–	–	24
Time per slice, ms	–	–	45.2
Echo spacing, ms	–	–	0.56
Field of view, mm			220
*N* volumes (A >> P)	–	–	112 + 1 *M*_0_
*N* volumes (P >> A)	–	–	1 *M*_0_
Acquisition time	–	–	6 min 44 s
**TRUST**			
Voxel, mm^3^	–	–	3.4 × 3.4 × 5
TR/TE, ms	–	–	3,000/7
Acceleration factor	–	–	GRAPPA = 3
Phase partial Fourier	–	–	6/8
Slab thickness/gap, mm	–	–	100/25
Echo spacing, ms	–	–	0.49
TI, ms	–	–	1,020
Flip angle, °	–	–	90
Field of view, mm	–	–	220
Acquisition time	–	–	1 min 27 sec

MRI: magnetic resonance imaging, TR: repetition time, TE: echo time, TI: inversion time, A: anterior, P: posterior, FLAIR: Fluid-attenuated inversion recovery, BOLD: blood oxygen level dependent, pCASL: pseudocontinuous arterial spin labelling, TRUST: T2-relaxation-under-spin-tagging MRI. *A subset of 145 out of the 552 participants at Wave MRI-1 on the Verio scanner also received pCASL scans (not vessel-encoded), the protocol for which is described in detail previously (Suri et al., 2019).

The study design was informed by Patient and Public Involvement (PPI), with a focus group of 5 participants including dementia support workers, patients, and carers. The study was piloted between July to September 2018, with 6 participants aged >65 years old, recruited through the Oxford Dementia and Ageing Research (OxDARE)^1^ database and mailing lists (CUREC Ethics Reference for pilot study: R58145). The PPI focus group and pilot were used to determine testing duration, feasibility and the protocols for the hypercapnic stimulus challenge.

### Selection of Participants

Postal invitations were sent to all 775 participants between July and October 2019. All participants undergo a telephone screening before their visit, during which exclusion criteria are evaluated, including contraindications to MRI (e.g., pacemaker, claustrophobia), respiratory contraindications which may affect their ability to perform a hypercapnic challenge (e.g., severe asthma, chronic obstructive pulmonary disease), and a clinical diagnosis of dementia.

Of the selection pool of 775, participants are excluded for Wave MRI-2 if they died or withdrew from the Whitehall II Study at Wave 13 or if they have gross incidental findings such as large strokes, tumours or cysts on their MRI-1 scan. Participants are prioritised for recruitment to Wave MRI-2 if they have complete information for key variables of interest in this study (APOE genotype, midlife vascular risk information at Waves 3 or 5, and complete T1-weighted, diffusion tensor imaging (DTI) and FLAIR scans at Wave MRI-1). Participants are also prioritised for recruitment if they were scanned on the Verio scanner at Wave MRI-1, as the Verio pool (a) received their baseline scans earlier on, allowing greater time to follow up, and (b) is larger, allowing us to reach the study’s initial target of *N* = 200.

Due to the COVID-19 pandemic, the *Heart and Brain Study* was temporarily paused in March 2020 after recruiting 22 participants. Analysis protocols developed with this data are presented in the following sections. The study will resume when risk assessments in line with national pandemic guidelines in the United Kingdom indicate it is safe to do so.

### Midlife Vascular and Genetic Risk for Dementia

Midlife vascular risk is assessed using composite risk scores calculated at Waves 3–11. Scores such as the Framingham Cardiovascular Disease Risk Score (FRS, comprising age, sex, body mass index (BMI), systolic blood pressure, total and HDL cholesterol, diabetes, smoking, antihypertensive treatment) (D’Agostino et al., 2008) and the CAIDE Dementia Risk Score (comprising age, sex, education, total cholesterol, BMI, systolic blood pressure, and physical activity) are used (Kivipelto et al., 2006). Both risk scores combine known risk factors for dementia and have been shown to predict cognitive decline and dementia within the Whitehall II cohort (Kaffashian et al., 2011) and other cohorts (Exalto et al., 2014). At Wave 3, approximately 29% and 34% of the MRI-1 cohort are classed as having moderate-to-high cardiovascular risk (FRS ≥ 10) and modifiable dementia risk (CAIDE ≥ 6), respectively.

Genetic risk is assessed as presence of the APOE4 allele, the best-established genetic risk variant for late-onset Alzheimer’s disease. APOE genotyping was performed at Wave 7 of the Whitehall II Study and the procedure has been described in detail elsewhere (Filippini et al., 2014). Of in the Wave MRI-1 cohort, 663 out of 775 participants have available APOE genotype information, 24.8% (157/633) are APOE4-carriers (defined as ε3ε4 or ε4ε4), 73.3 % are non-carriers (defined as ε3ε3, ε2ε3, and ε2ε2), and 1.8% are ε2ε4 carriers.

### 3T Brain MRI

Magnetic resonance imaging scans for the *Heart and Brain Study* are acquired using a 3T Siemens Prisma scanner at the OHBA. Scans are processed using FMRIB Software Library (FSL) (Jenkinson et al., 2012) and FreeSurfer (Fischl et al., 2002; Reuter et al., 2012), and visually inspected in FSLeyes (McCarthy, 2020), with reference to pipelines adapted from the Whitehall II Imaging Sub-study and the UK Biobank Study (Alfaro-Almagro et al., 2018)^2^. To reduce spatial distortions, a gradient distortion correction (GDC) is applied to scans^3^ using a proprietary Siemens data file that describes gradient non-linearities. To protect participant anonymity, all high-resolution scans are “defaced” to remove the eyes, nose, mouth and ears using the “fsl_deface” tool. Image acquisition parameters at Waves MRI-1 and MRI-2 are presented in Table 2.

#### Structural Imaging

##### T1-weighted imaging

T1 scans provide information about tissue morphology and volumes and are an essential step in the processing of almost all other MRI modalities. A 1 mm isotropic 3D MPRAGE scan is acquired using the “pre-scan normalise” option to perform partial bias field correction during acquisition (van der Kouwe et al., 2008). At both the MRI-1 and MRI-2 waves, GDC-corrected and defaced images are re-oriented to MNI152 space, cropped to reduce the amount of non-brain tissue, bias-field corrected, registered to the 1 mm standard MNI152 space using non-linear registration, and brain-extracted using the inverse of the MNI152 alignment warp to generate a brain-extracted T1. All of the above is performed using the automated FSL-ANAT tool. The pre-processed images are then segmented to produce partial volumes for total GM, WM, cerebrospinal fluid (CSF), and sub-cortical regions. Subsequently, volumes can be extracted in mm^3^ and normalised as percentages of total brain (GM+WM) volume and total intracranial volume (GM + WM + CSF). As the T1 acquisitions at Wave MRI-2 have brighter carotid arteries than those at MRI-1, we perform an additional “intensity clipping” step for harmonization. Briefly, we create a custom brain mask that excludes these arteries before estimating regional and local volumes.

To investigate longitudinal atrophy between Waves MRI-1 and MRI-2, established pipelines which incorporate additional temporal information will be used. These include the FreeSurfer Longitudinal Pipeline (Reuter et al., 2012), which estimates reliable changes in volume and thickness for cortical and subcortical regions; the FSL-SIENA tool which estimates percentage brain volume change between two time points (Smith et al., 2002), and the modified voxel-based morphometry pipeline to assess voxel-wise microstructural changes (Chételat et al., 2005).

##### Diffusion tensor imaging

Diffusion tensor imaging is sensitive to the anisotropic diffusion of water within the axon, i.e., the diffusion is unrestricted along the axon, but hindered perpendicularly due to the myelin sheath. The directionality and magnitude of water diffusion is quantified by DTI-derived metrics such as fractional anisotropy (FA), radial diffusivity (RD), axial diffusivity (AD), and mean diffusivity (MD). Decreases in FA alongside increases in diffusivity are well-established measures of WM impairments in dementia (Suri et al., 2014b).

Diffusion tensor imaging scans are processed as described for Wave MRI-1 and the UK Biobank Study. Briefly, scans are analysed using the FMRIB diffusion toolbox (Smith et al., 2006), which performs susceptibility-induced distortion correction using FSL-TOPUP, brain extraction on the distortion-corrected B0 image using FSL-BET, and motion and eddy current correction using FSL-EDDY (Behrens et al., 2003). A single reverse (posterior-to-anterior) phase-encoding non-diffusion weighted (*b*-value = 0 s/mm^2^) volume is acquired, to generate appropriate fieldmaps for the “topup” tool. The resulting aligned and distortion corrected diffusion images are fed into “dtifit”, which fits a diffusion tensor model at each voxel and produces FA and diffusivity maps. These maps are then entered into the TBSS pipeline to perform microstructural analysis across subjects. FA maps are aligned to standard FMRIB58_FA space using FNIRT and thinned to create a mean FA skeleton representing the centres of all WM tracts common to the group. This is then repeated for MD, RD and AD. Measures of global FA, MD, RD and AD can then be extracted from the mean skeleton, and the respective subject-specific spatial maps can be concatenated and entered into voxel-wise statistics using the FSL-randomise tool.

#### Cerebrovascular Imaging

##### Cerebrovascular reactivity

In this study, CO_2_-CVR is measured as the compensatory change in the BOLD signal in response to a 5% CO_2_ challenge. The respiratory paradigm consists of 60 s of air followed by two 75 s blocks of hypercapnia interleaved with two 75 s blocks of air. Multi-band BOLD fMRI scans are acquired every 800 ms during the respiratory challenge (see Table 2 for acquisition parameters). Participants are asked to lie still with their eyes open, and to breathe normally during the scan as both posture (Favre et al., 2020) and eye opening (Peng et al., 2013) have been shown to affect the BOLD response.

**FIGURE 2:**
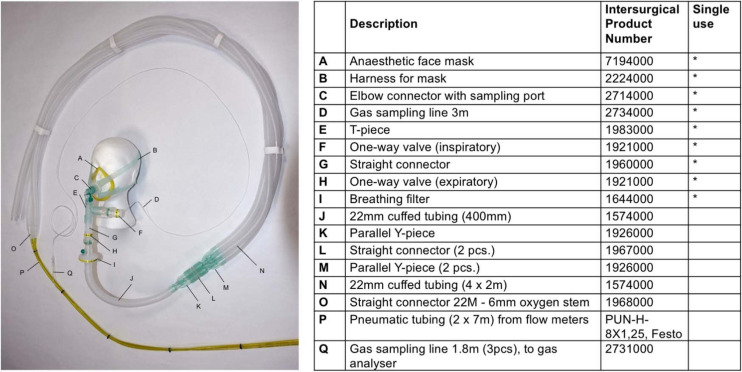
Breathing circuit for the CO_2_-CVR respiratory paradigm, and the corresponding product identification numbers from Intersurgical Ltd., Wokingham, United Kingdom.

Inspired gases are delivered to a face mask at a rate of 15 L/min via a unidirectional breathing circuit designed in-house at the University of Oxford using parts from Intersurgical Ltd., Wokingham, United Kingdom (Figure 2). A gas analyser (ML206, ADInstruments, New Zealand) and data acquisition system (PowerLab 4/35, ADInstruments, New Zealand) are connected to the face mask via a sampling line and used to obtain continuous recordings of inspired and expired oxygen and CO_2_ concentration throughout the scan. This produces a full respiratory trace from which end-tidal values are subsequently obtained.

The gas delivery system consists of a cylinder of synthetic medical air (191-J, BOC Limited, United Kingdom) and one of 5% CO_2_ in medical air (299034-L-PC, BOC Limited, United Kingdom), each fitted with a pressure regulator (Gasarc Tech-Master, ESAB, United Kingdom) and connected to a variable area flow meter (99-0377, The Gas Safety Co, United Kingdom) via a solenoid valve (Type 6013, Bürkert, Germany). The valves are operated by an automated valve controller designed in-house, programmed with the respiratory paradigm, and triggered directly by the MRI scanner. From the flow meters, the gases are piped into the breathing circuit shown in Figure 2.

The breathing circuit consists of a disposable anaesthetic face mask placed over the participant’s mouth and nose and fastened with a harness. The mask is connected to a disposable elbow piece with a sampling port linked to the data acquisition system, allowing gas concentrations to be measured continuously. The elbow piece also connects to a disposable T-piece, with a one-way outlet valve on one leg (providing an unimpeded path for expired air) and a one-way inlet valve on the other leg (supplying the inspired gas). A medical grade breathing filter is placed at the junction of the disposable circuitry and the fixtures, to prevent cross-contamination of the fixtures. The circuit is open to room air via an additional length of tubing that serves as a gas reservoir. This guarantees that participants are safely able to continue breathing if the gas flow is unexpectedly interrupted or insufficient.

In order to familiarize participants with the hypercapnia challenge and check for leaks in the breathing circuit, we briefly deliver the gases to participants for a test period before they enter the MRI scanner. Leaks at the mask-face interface identified via the capnometry trace are plugged with additional rubber fittings if needed. During the scan, heart rate, and respiratory effort are monitored non-invasively using a pulse plethysmograph and chest belt. The participant’s respiratory trace is observed for indications of stress, and they are provided with a buzzer to request immediate removal from the scanner at any point.

**FIGURE 3:**
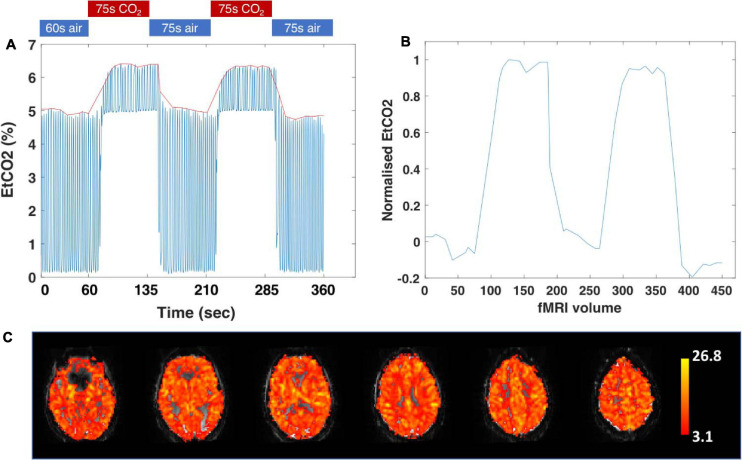
Analysis of CVR scans. **(A)** The 6-min end-tidal CO_2_ (EtCO_2_) capnometry trace for a *Heart and Brain Study* participant. Each blue peak represents one breath and the red line follows the EtCO_2_ at expiration, resampled at the TR. Participants inhaled either normocapnic air (0.04% CO_2_) or air mixed with 5% CO_2_ through a face mask. For the first 60 s participants inhaled air, followed by two alternating 75 s blocks of 5% CO_2_ in air, and air. MRI data was collected from 0s. **(B)** Normalised and averaged EtCO_2_ at each TR, used as an explanatory variable in fMRI analysis. **(C)** Thresholded activation images, showing a BOLD signal change during the hypercapnia blocks relative to air. Red to yellow colours represent 3.1 < *z*-statistic < 26.8.

Figure 3 describes the data analysis for a single participant. Each participant’s respiratory trace is processed using an in-house MATLAB script (Bulte et al., 2012; Suri et al., 2014a) which extracts the end-tidal CO_2_ (EtCO_2_) values, then interpolates, trims, shifts and resamples the data to align with the BOLD time course and TR. This produces one EtCO_2_ value per fMRI volume. These data are then normalised to use as a regressor. The raw fMRI data is processed with FSL-FEAT, which performs motion correction, brain extraction, spatial smoothing (here, using a kernel of 4 mm), high-pass temporal filtering (here, using a cut-off 210 Hz, representing one 60s baseline block + one 75s hypercapnia block + one 75s normocapnia block), and distortion correction using fieldmaps processed with the “fsl_prepare_fieldmap” tool. The normalised EtCO_2_ values are entered as explanatory variables in FEAT, which uses general linear modelling to fit the experimental design to the associated BOLD time-course. This ultimately highlights the brain regions which show a response to the hypercapnic stimulus. For each subject, mean CVR is then extracted from regions of interest using Featquery, and expressed as the percentage change in BOLD signal per mmHg change in EtCO_2_ (%BOLD/ΔEtCO_2_ mmHg), where the total change in EtCO_2_ is the difference between the mean baseline value and the mean of the maximum change in the two hypercapnic periods. The processed fMRI images can also be registered to standard MNI152 space and submitted for cross-subject voxel-wise statistics using higher-level FEAT analyses.

##### Feasibility and tolerability of CO_2_-CVR scan

**FIGURE 4:**
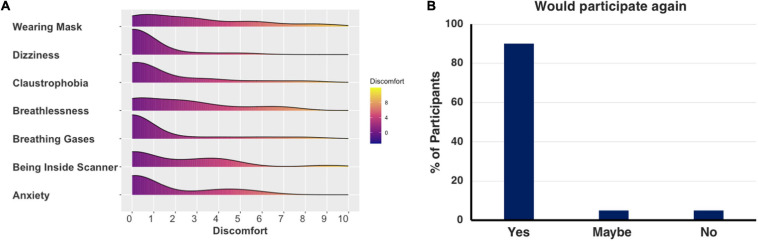
Tolerability of the CO_2_-CVR scan. Plots show responses from 21 participants of the *Heart and Brain Study* stating **(A)** the level of discomfort (rated from 0 to 10) related to the different symptoms during a 5% CO_2_ challenge and **(B)** the willingness to participate in a similar respiratory-calibrated MRI study again. Responses suggest that a 6-minute respiratory paradigm with a 5% CO_2_ challenge is generally well-tolerated and feasible in older adults.

Immediately after their MRI scan, participants complete a questionnaire to rate their experience of breathlessness, anxiety, claustrophobia and discomfort during the respiratory paradigm on a scale of 0–10 (0–3 low discomfort, 4–7: moderate discomfort, 8–10: severe discomfort). The full questionnaire is presented in the Supplementary Materials and was developed together with the Imaging Cerebral Physiology Network^4^. Of the 22 participants scanned thus far, one participant did not complete the questionnaire due to time constraints, and responses from the remaining 21 participants are presented in Figure 4. Overall, 24% (5/21) of participants noticed when they were breathing different gases, but only 5% (1/21) noticed a change in smell or taste. In an open-ended question about the least comfortable part of the CVR scan, 43% (9/21) participants mentioned general scanner-related sources of discomfort such as scanner noise and difficulty lying still or staying awake, 43% (9/21) participants mentioned feeling restricted or breathless due to the mask and gases, and 14% (3/21) stated that they experienced no discomfort. Importantly, when asked whether they would be willing to participate in a similar study again, 90% (19/21) said “Yes”, 1 participant said “Maybe” and only 1 answered “No”, due to mask-related claustrophobia. Taken together these responses suggest that a 6-min respiratory paradigm with a 5% CO_2_ challenge is generally well-tolerated and feasible in adults aged > 65 years old, who have a range of risk factors for dementia.

##### Vessel-encoded cerebral perfusion (pseudocontinuous arterial spin labelling)

Cerebral blood flow is typically measured non-invasively using pseudocontinuous arterial spin labelling (pCASL), which quantifies total perfusion as the rate of delivery of arterial blood to brain tissue (millilitres of blood per 100 g of tissue per minute) (Alsop et al., 2015). Recent advances in pCASL imaging offer additional vessel-specificity, so that in addition to obtaining measures of total CBF, we can also image and quantify perfusion from each of the four major arteries supplying the brain, *viz.* the right and left internal carotid arteries and vertebral arteries. Vessel-encoded pCASL has the added advantage of assessing (a) variations in vascular territories in individuals with differing dementia risk (b) compensatory collateral flow as a result of underlying vascular disease, which is not otherwise possible with pCASL scans of total cerebral perfusion (Okell et al., 2013; Jezzard et al., 2017). Vessel-encoded scans also provide more accurate perfusion estimates in cases where there is mixed blood supply, and the arterial arrival time from each artery differs.

We acquire a multi post-labelling delay (PLD) vessel-encoded pCASL scan with a labelling duration of 1.4 s, and 7 PLDs (0.25, 0.50, 0.75, 1.0, 1.25, 1.5, and 1.75 s) with two averages of 8 vessel-encoding cycles at each PLD (i.e., 7 PLDs × 8 encoding cycles × 2 averages for each PLD/encoding pair = 112 volumes). A 3D multi-slab time-of-flight sequence is acquired immediately prior to the pCASL scan to enable localisation of the labelling plane and vessels. The four arteries are labelled in different combinations during acquisition, as described previously (Okell et al., 2013), and the contribution of each artery to the resulting signal is decoded in post-processing using the VEASL tool within the BASIL (Bayesian Inference for Arterial Spin Labelling) toolkit (Chappell et al., 2009, 2010, 2012). BASIL uses a variational Bayes approach to perform a nonlinear fit of the general kinetic model to the pCASL data for all voxels in the brain. One *M*_0_ calibration scan is acquired to calibrate the pCASL perfusion-weighted signal by estimating the equilibrium magnetization of blood, and a single reverse phase-encoded (P >> A) calibration scan with the same parameters is acquired for distortion correction using the FSL “topup” tool (Andersson et al., 2003). The analysis of vessel-encoded pCASL data is similar to that of pCASL scans (Suri et al., 2019) with an additional step to decode the vessel contributions after pre-processing, and fitting the kinetic model to each vessel component separately. The vessel decoding process has been described in detail previously (Chappell et al., 2010; Okell et al., 2013).

**FIGURE 5:**
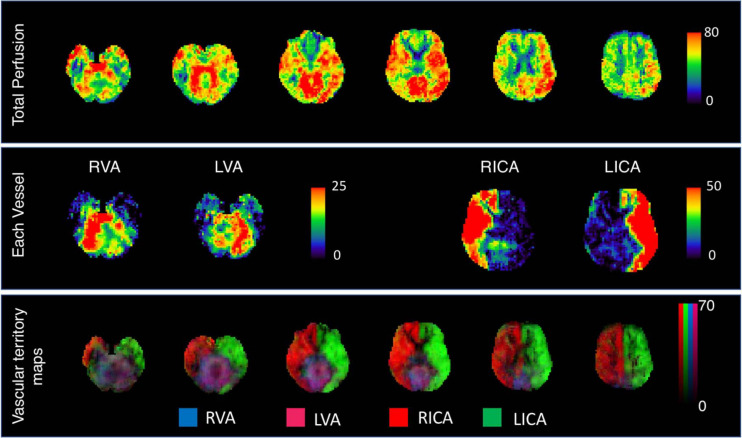
Vessel-encoded pCASL data from a *Heart and Brain Study* participant. The top panel displays total cerebral perfusion in ml/100 g tissue/min across six horizontal slices in native ASL space. Warmer colours representing regions of higher perfusion. The middle panel displays perfusion from each of the four encoded vessels for a single slice (RVA, LVA: right and left vertebral arteries, RICA, LICA: right and left internal carotid arteries). The bottom panel displays combined vascular territory maps where signals from each of the four feeding arteries are summed (green: LICA, red: RICA, blue: RVA, and magenta: LVA).

Figure 5 displays the vessel-encoded pCASL output from a single participant at Wave MRI-2. A file containing the X and Y coordinates of the initial locations of the four encoded vessels is generated from the DICOM perfusion data. Raw scans are acquired using the “Pre-scan normalise” option and pre-processed using the “oxasl” tool, with which the images are averaged, decoded, and used to generate artery-specific perfusion maps. Recommended fixed values for T1 of tissue (1.3s) and blood (1.65s) are used (Alsop et al., 2015). The arterial transit time for each feeding artery is estimated voxel-wise from the data assuming a prior mean of 1.3 s, as per previous studies (Okell et al., 2013; Harston et al., 2017). A weighted whole-brain arterial transit time map is calculated can then be calculated as described previously and used for cross-subject voxel-wise statistics (Okell et al., 2013). Slice timing correction (an increase in PLD of 0.0452s per slice) is applied (Groves et al., 2009). The T1 scans are used to automatically define the CSF in the ventricles and the calibration image is used to calculate the equilibrium magnetization of ventricle CSF in the ventricles. This is converted to the equivalent value in arterial blood (accounting for differences in proton density) and used to give perfusion values in absolute units of ml/100 g/min for each vessel. The output of the individual vessels is summed to give total cerebral perfusion in each voxel in native ASL space.

To run cross-subject voxel-wise statistics, ASL scans are registered to standard MNI152 space. Partial volume effects can arise from the typically low spatial resolution of pCASL data and are a potential confounding factor given the tissue-specific kinetics (WM tends to have lower CBF and longer arrival times than GM). This can be particularly complicated in populations with age-related atrophy, thus an automatic partial volume correction will be applied (Chappell et al., 2011). This uses high-resolution partial volume estimates from the structural image to produce separate GM and WM perfusion maps in native and standard space. Results with and without partial volume correction will be analysed.

##### White matter hyperintensities

FLAIR scans provide information on white matter hyperintensities (WMHs), a key pathology of vascular and Alzheimer’s dementia (Wardlaw et al., 2015). Here, WMHs are quantified using the FSL-BIANCA tool, a fully automated, supervised tool for WMH segmentation, based on the k-nearest neighbour algorithm (Griffanti et al., 2016). BIANCA has been optimised on two clinical datasets, applied in healthy older adults (Griffanti et al., 2018), and trained using an openly available training dataset (“Mixed_WH-UKB_FLAIR_T1”, available at^5^, Bordin et al., 2020). The training dataset was generated using FLAIR, T1 and manually segmented WMH images from a sub-sample of 24 participants each from the Whitehall II Imaging Sub-study Siemens Verio 3T scanner, the Prisma 3T scanner, and 12 participants from the UK Biobank Study (Siemens Skyra 3T). Training BIANCA with this dataset reduces the variability in BIANCA performance and generates more consistent WMH measures across images acquired in different cohorts and scanners (Bordin et al., 2020).

To quantify the WMH load, we first create a WM mask in T1 space, which excludes cortical and subcortical GM (details in Griffanti et al. (2016)). This mask, together with bias-corrected and brain-extracted T1 images and T1 brain masks, is then linearly registered to FLAIR space using FLIRT. The GDC-corrected and defaced FLAIR scans are brain-extracted (by applying the registered T1 brain mask) and bias-corrected. They are then masked using the WM mask generated in the first step in order to reduce the detection of false positive hyperintensities from cortical and subcortical GM. For each subject, BIANCA is run using the masked FLAIR image, the brain-extracted T1 image and a FLAIR-to-MNI transformation matrix. This pipeline classifies voxels by their intensity and spatial features, producing a map with the probability of each voxel being a WMH. The probability maps are subsequently thresholded at 0.8 or 0.9 based on a visual assessment of WMH load (higher WMH load requires lower thresholds) and then binarized. WMH volumes are then extracted in mm^3^ and expressed as % of total brain volume (WM + GM) and total intracranial volume (WM + GM + CSF).

##### Evaluation of BIANCA performance across scanners

Although the same FLAIR sequence was used at both the MRI waves and a harmonised training dataset was used, we performed further evaluations of BIANCA to ensure that it was performing equally well on images from the scanners at MRI-1 (3T Verio) and MRI-2 (3T Prisma) (Griffanti et al., 2016). We created manually segmented WMH masks for 12 participants of the *Heart and Brain Study*, who had a range of WMH loads at Waves MRI-1 and MRI-2. We then measured the volumetric agreement between the total WMH volume obtained from BIANCA and manual segmentations using the intra class correlation coefficient (ICC; two-way mixed model with absolute agreement definition). BIANCA performance was also evaluated by testing the interaction between manually segmented WMH volumes (independent variable) and MRI wave on the Dice Similarity Index, DSI (dependent variable; a summary measure of overlap between manual and BIANCA segmentations). Similar regression slopes (i.e., no interaction between MRI wave and WMH volumes) and regression intercepts (i.e., no main effect of MRI wave) would indicate successful between-scanner harmonisation.

(1) $$\text{DSI}=\frac{\begin{array}{c} 2\times (\mathrm{voxels}\mathrm{in}\mathrm{the}\mathrm{intersection}\mathrm{of}\mathrm{manually}\\ \mathrm{segmented}\mathrm{and}\mathrm{BIANCA}\mathrm{segmented}\mathrm{masks}) \end{array}}{\begin{array}{c} \mathrm{voxels}\,\mathrm{in}\,\mathrm{the}\,\mathrm{manual}\,\mathrm{mask}+\\ \mathrm{voxels}\,\mathrm{in}\,\mathrm{the}\,\mathrm{BIANCA}\,\mathrm{mask} \end{array}}$$

**FIGURE 6:**
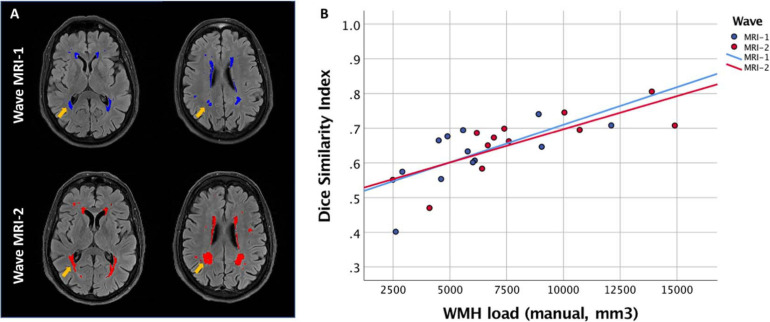
White matter hyperintensity (WMH) lesion load. **(A)** Data from *Heart and Brain Study* participant showing automatically segmented WMH at Wave MRI-1 (2012-16, blue) and Wave MRI-2 (2019-23, red), using BIANCA. Yellow arrows indicate regions with noticeable increases in WMH between the two waves. **(B)** Scatter plot of BIANCA performance for 12 subjects, demonstrating that BIANCA performs similarly across the two timepoints. The Dice Similarity Index and manually segmented WMH load (mm^3^) are plotted on the *Y* and *X* axes, respectively.

Overall, there was an excellent agreement between manual and BIANCA segmentations at Wave MRI-1 (ICC [95% CI] = 0.97 [0.87, 0.99]) and Wave MRI-2 (ICC [95% CI] = 0.98 [0.66, 0.99]). The volume in mm^3^ of WMHs was significantly higher at MRI-2 than in MRI-1, for both the manual segmentations (paired *t*(11) = 4.7, difference of the means [95% CI] = 2,024.1 [1,065.1–2,983.0], *p* < 0.001) and automatic BIANCA segmentations (*t*(11) = 5.1, difference of the means [95% CI] = 2,447.9 [1,396.6–3,499.2], *p* < 0.001). Despite the increase in the WMH load, BIANCA performed similarly across both the timepoints and scanners, as evidenced by (a) the overlapping linear regression lines at the MRI-1 and MRI-2 waves (Figure 6), (b) no main effect of MRI wave on the DSI [*B*(SE) = 0.012 (0.07), *p* = 0.85], and (c) no significant interaction between MRI Wave and WMH volumes [*B*(SE) = −2.5e-06 (8.9e-06), *p* = 0.78].

##### Cerebral venous oxygenation

Venous oxygenation is the fraction of oxygenated haemoglobin in the venous blood. Measurements of venous oxygenation will be used to estimate the global oxygen extraction fraction (OEF) and global cerebral metabolic rate of oxygen consumption (CMRO_2_) which are key determinants of the BOLD signal. Here, we use T_2_-Relaxation-Under-Spin-Tagging (TRUST) MRI scans to non-invasively quantify blood oxygenation in the brain, and the theoretical frameword for this sequence has been described in detail elsewhere (Lu and Ge, 2008; Xu et al., 2012; Jiang et al., 2018). The pulse sequence of this scan is based on the pulsed ASL technique. The labelling slab is placed above the imaging slice to label venous blood. The PLD is optimised to allow sufficient blood to be delivered to a slice placed perpendicular to the sagittal sinus.

Scans are processed using in-house MATLAB scripts, which perform motion correction and pair-wise subtraction of the control and label images. A region of interest is drawn around the superior sagittal sinus, within which four voxels with the highest blood signals (according to the difference signals) are selected. The spatial average signal from these voxels is then fitted to an exponential function of the effective echo time to calculate blood R_2_ (transverse relaxation rate) assuming the *T*_1_ of blood as 1,624 ms. Venous oxygenation is then estimated from the blood *R*_2_ using a calibration curve and assuming haematocrit = 0.42, as described previously (Xu et al., 2012). OEF is expressed as a percentage (%) of the oxygen in arterial blood extracted to serve oxidative metabolism.

### Cardiometabolic Measurements

Following a brief period of rest, participants receive measurements of body mass index (BMI) and blood pressure.

#### Body Mass Index

Height (cm) and weight (kg) are measured to derive BMI as weight/height^2^.

#### Peripheral Pressure

Peripheral blood pressure is measured using an automated sphygmomanometer, while the participant is seated (OMRON HEM-907; OMRON Healthcare UK Ltd., Milton Keynes). Two measurements each of pulse rate, systolic pressure (SBP) and diastolic pressure (DBP) are made from the right arm and averaged. Pulse pressure is derived as SBP – DBP and mean arterial pressure as ([SBP] + 2[DBP])/3.

#### Central Blood Pressure

Pulse wave analysis (PWA) is used to estimate central blood pressure at the level of the aorta. For this technique, a tonometer (SphygmoCor MM3, AtCor Medical, Australia) is used to record a peripheral pressure waveform from the right radial artery, while the participant is seated. The peripheral pressure waveform is used with a generalised transfer function to derive aortic SBP, pulse pressure and other PWA parameters (Pauca et al., 2001). Consistency of the waveform is assessed during acquisition, and recordings are repeated if quality control criteria (i.e., operator index ≥ 80) are not met.

### Vascular Sonography

Long-term exposure to vascular risk can progressively stiffen the aorta and other elastic arteries. The aorta branches into the common carotid artery, which in turn bifurcates in the neck to form the internal and external carotid arteries (which supply the brain and rest of head, respectively). Here, ultrasound scans are performed of the ascending aorta and left and right common carotid arteries to qualify and quantify large artery phenotypes such as vessel stiffness, thickness and wave transmission. Scans are performed on a GE VIVID 7 system, in a room with dimmed lighting. Participants are allowed 10 min of rest prior to the scan and ECG traces are recorded concomitantly with the ultrasound acquisitions.

#### Left and Right Common Carotid Arteries

**FIGURE 7:**
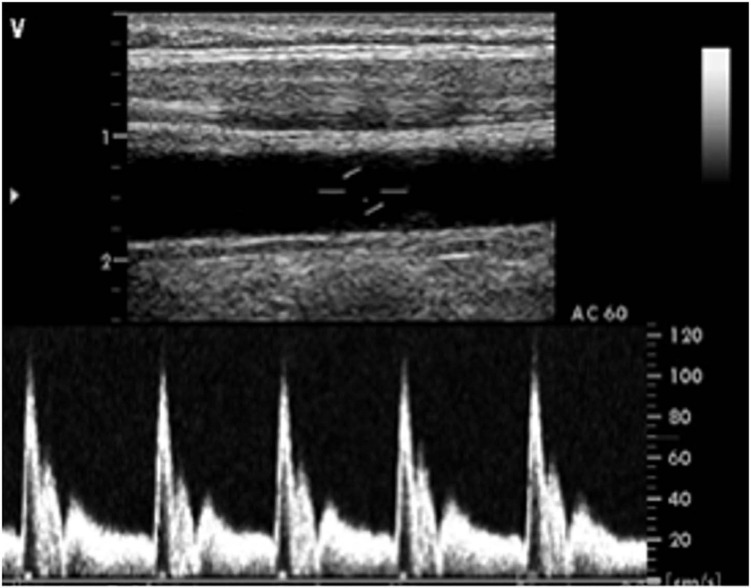
Vascular duplex sonography scan of the left carotid artery showing the longitudinal image of the artery **(top)** and the corresponding Doppler pulse wave velocity trace **(bottom)**.

Measurements are performed using an M12L linear array transducer (centre frequency 14.0 MHz) while participants are rested in the supine position. First, longitudinal B-mode images of the common carotid artery are taken in the ear-to-ear plane at a location 1–2 cm proximal to the carotid bifurcation. Carotid wall motion is tracked for ∼20–30 heart beats and saved as a DICOM file in order to allow offline calculation of vessel diameter, compliance, distensibility, and β stiffness index using specialist software (CaroLab 5.0, Zahnd et al., 2019). Next, duplex Doppler images are taken in the same location for a further 20–30 heartbeats, with the beam insonation angle maintained ≤60° in order to allow the estimation of the blood velocity waveform (Figure 7), from which the volumetric blood flow can be estimated using the measured diameter.

Using custom-designed algorithms (Negoita et al., 2018a) using MATLAB, R2019B, The MathWorks, MA), local carotid artery wave speed (c) and wave intensity (WI) are then determined in order to provide further insights into vessel stiffness and to characterise differences in wave transmission to-and-from the cerebral circulation. Full details of these techniques and their clinical applicability have been published elsewhere (Sugawara et al., 2009; Borlotti et al., 2012). In brief, WI analysis provides a non-invasive measure of the net energy density flux carried by forward- and backward-travelling waves within the arterial system. During each cardiac cycle, left ventricular contraction results in the generation of a large forward-travelling compression wave (FCW) during early systole which causes blood velocity and pressure to increase in tandem. Shortly afterwards, a reflected backwards-travelling compression wave (BCW) is commonly apparent during mid-systole, resulting in a slowing of velocity while continuing to increase pressure. Finally, a forward-travelling expansion wave (FEW) generated by the onset of left ventricular relaxation is observed at the end of systole. Here, the FCW and BCW will be our primary WI outcomes of interest, as these have been shown to provide information on risk of cognitive decline and cerebrovascular vasomotor tone, respectively (Bleasdale et al., 2003; Chiesa et al., 2019).

The non-invasive determination of the local wave speed (c) can be expressed as previously described (Feng and Khir, 2010):

$$\text{c}=\pm \frac{1}{2}\,\frac{d\,U_{\pm }}{d\,l\,n\,D_{\pm }}$$

where dlnD and dU represent simultaneous logarithmic changes in vessel diameter and change in blood flow velocity, respectively. The local wave speed (c) which – assuming that reflected waves are absent during the earliest part of systole – can be calculated from the slope of the initial linear part of a lnDU loop.

The net WI waveform (dI) waveform can be calculated as,

dI=dD.dU

The net WI can be further separated into its forward (+) and backward (−) travelling components using the equation:

dI=±±14⁢(D2⁢c)(dD±D2⁢cdU)2

where +/− indicate forward and backward travelling direction of the wave.

#### Ascending Aortic Artery

In addition to common carotid arteries, similar measurements are also recorded from the ascending aorta using a 7S array transducer (frequency 6.0 MHz), while the participant is in the left lateral decubitus position. First, ∼20–30 heartbeats of continuous M-mode data are measured in the parasternal long axis view in order to visualise changes in vessel diameter over time, from which vessel distensibility can be calculated. Subsequently, blood velocity is measured over a similar timeframe in the apical five-chamber view using pulse-wave Doppler, and custom-built MATLAB algorithms are once again employed to trace the velocity waveform. The velocity and diameter waveforms are then used to calculate local wave speed and WI, as previously shown in the ascending aorta (Negoita et al., 2018b).

### Cognitive Battery

In order to evaluate longitudinal change in cognitive performance between the two MRI waves, a subset of the same tests used at Wave MRI-1 are administered in the same order at Wave MRI-2. The tests have been extensively validated and shown to be sensitive to cognitive impairment.

#### Montreal Cognitive Assessment

A 30-point cognitive screening test assessing visuo-spatial abilities (4 points), short-term memory (5 points), executive function (3 points), attention and working memory (6 points), language and phonemic fluency (6 points), and orientation (6 points). Participants receive an additional point if they have fewer than 12 years of full-time education (Nasreddine et al., 2005).

#### Trail Making Test Versions A and B

An executive function and processing speed task where participants connect a series of 25 circles first in ascending numerical order (TMT-A) and then in alternating alphabetical and numerical order (TMT-B) (Lezak et al., 2004).

#### The Hopkins Verbal Learning Test-Revised

A verbal learning and episodic memory task where participants learn twelve words over three trials and recall and recognise them immediately and after a delay of 30–45 min (Brandt, 1991).

#### Digit Span Test, From Wechsler’s Adult Intelligence Scale

A working memory task where participants immediately recall a list of numbers read to them in the forwards, backwards and ascending order. The number of digits in each sequence gradually increases until the participant fails or reaches a maximum score (Wechsler et al., 2008).

#### Category Fluency Test, From Addenbrooke’s Cognitive Examination Revised

A semantic memory task where participants list as many animals as possible within 60 s (Mioshi et al., 2006).

#### Rey Complex Figure Test and Recognition Trial

A visuo-spatial memory, working memory, planning and attention task, where participants copy and then recall a complex geometric diagram immediately and after a delay of 30–45 min (Meyers and Meyers, 1995).

#### Boston Naming Test

A semantic memory task, where participants name a series of 60 images of increasing difficulty (Kaplan et al., 1983).

#### Digit Coding Test, From the Wechsler Adult Intelligence Scale – Fourth Edition

A working memory and executive function task where participants have to write the appropriate novel symbol for each number within a given time (2 min) (Wechsler et al., 2008).

#### Test of Premorbid Functioning

A premorbid IQ test where participants read aloud a list of 70 written words. It is marked according to pronunciation and used to estimate intellectual functioning before disease onset (Wechsler, 2011).

### Self-Administered Clinical Questionnaire

The following self-report questionnaires which were completed at Wave MRI-1 (and described in detail in Filippini et al. (2014)) are completed by participants in the week prior to their testing appointment for Wave MRI-2.

#### Psychiatric and Psychological Questionnaires

The General Health Questionnaire-30 for detection of psychiatric illness in community settings (Goldberg, 1988), Mood Disorder Questionnaire for assessment of bipolar disorders (Hirschfeld, 2002), the Centre for Epidemiological Studies Depression (CESD) Scale for the assessment of major depressive symptomatology (Radloff, 1977), the State and Trait Anxiety Inventory to assess current and general anxiety (Spielberger, 1989), Penn State Worry Questionnaire (ultra-brief version) to assess pathological worry (Berle et al., 2011), MacArthur Stress Reactivity Questionnaire to assess reactions to stressful situations (Taylor and Seeman, 1999), the 5-Dimensional Curiosity Scale to gauge the desire to seek novel experiences (Kashdan et al., 2018).

#### Lifestyle and Life Experience Questionnaires

CHAMPS Physical Activity Questionnaire for Older Adults to assess weekly frequency and duration of exercise (Stewart et al., 2001), the Locus for Causality Exercise Questionnaire to assess motivation to exercise (Miller et al., 1988), Pittsburgh Sleep Quality Index (Buysse et al., 1989) and Jenkins Sleep Questionnaire (Jenkins et al., 1988) to assess sleep quality in the month prior to their visit, Life-Orientation Revised Questionnaire to gauge optimism for future events (Scheier et al., 1994), Life Events questionnaire to report past stressful experiences (Brugha and Cragg, 1990), and a Handedness questionnaire to assess preferences for right or left-handedness (Briggs and Nebes, 1975). Participants also provide information on the frequency of smoking and alcohol intake.

#### Demographics and Medical History

Participants provide information on age, years of education, longstanding illnesses, hospitalizations, current or past medications, and diagnoses or self-report of diseases.

## Discussion

**FIGURE 8:**
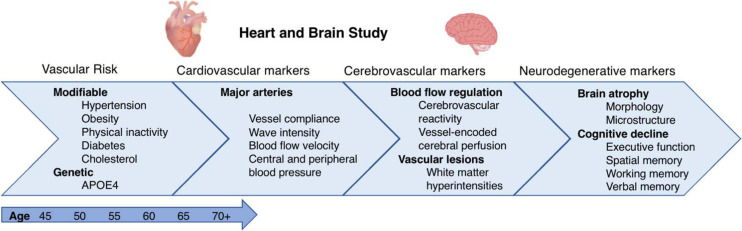
Mechanistic framework for the *Heart and Brain Study*. The study will examine how 30-year trajectories of vascular risk throughout midlife (40–65 years) affect cardiovascular and large artery phenotypes, cerebrovascular health, longitudinal brain atrophy and cognitive decline at older ages (>65 years). The moderating role of genetic risk (APOE4) will also be examined.

The *Heart and Brain Study* will integrate trajectories of risk over 30 years, with longitudinal brain ageing and neuropsychiatric measures, and novel cerebrovascular and cardiovascular imaging markers. The overarching goal of this study is to identify and understand key modifiable and genetic determinants of risk and resilience for brain and cognitive ageing, with a primary focus on the heart–brain axis. The following analyses were proposed when applying for funding and will be investigated in this study (Figure 8):

a) Disturbances in haemodynamics and biomechanical properties of the aorta and systemic large elastic arteries will be associated with cerebral and cognitive health. Several studies have linked aortic stiffness with dementia and cognitive decline (for review see Iulita et al. (2018)), and we recently noted that faster aortic stiffening is also related to poor brain microstructural, perfusion and cognitive outcomes (Suri et al., 2020). Here we will additionally examine whether this association may result from haemodynamic changes in the carotid arteries. For example, investigating the association between circulation in the common carotid arteries with cerebral perfusion from the internal carotid arteries would enable direct inferences of how large artery circulation affects the cerebral microvasculature.
b) Potentially modifiable vascular risk factors for dementia will be associated cardio- and cerebrovascular outcomes (viz. aortic and carotid phenotypes, CVR, cerebral perfusion, WMHs, and cerebrovascular lesions) in older age. Importantly, 30-year trajectories of vascular risk will be assessed to determine the stage of life when their effects on brain health may be most pronounced. The moderating role of APOE4 in these associations will also be examined.
c) Cross-sectional MRI studies have reported associations between CVR and cognitive impairments (for review see Catchlove et al., 2018), and there is a well-established link between reduced total CBF and dementia (Iadecola, 2004). However, to date, their relationship with longitudinal brain atrophy and cognitive decline remain poorly understood. Here, the associations of CVR and vessel-specific CBF with two time-point changes in vascular lesions (e.g., WMHs), cerebral morphology (e.g., atrophy of key areas such as the hippocampus), microstructure (e.g., changes in FA and diffusivity) and cognitive decline (e.g., verbal memory, executive function, spatial memory) will be examined.

The *Heart and Brain Study* will generate one of the most comprehensive datasets to study the longitudinal determinants of the heart–brain axis in ageing. The study will investigate novel physiological mechanisms in this pathway, with a view to describing the optimal window for managing vascular risk in order to delay cognitive decline. We will evaluate imaging markers for potential use in clinical trials and inform strategies to identify at-risk individuals for targeted interventions to prevent or delay dementia. In addition, our efforts will contribute to the advancement and validation of MRI analysis and harmonisation methods for longitudinal neuroimaging cohorts. Thus, information gained from this extensively characterised and long-running cohort will supplement ongoing larger-scale projects such as the UK Biobank Study. Based in the Department of Psychiatry and Wellcome Centre for Integrative Neuroimaging at the University of Oxford, the proposed work will involve close collaboration with neuroimaging, vascular imaging, MRI physics and analysis, dementia research, and epidemiology experts at University College London, Brunel University London, and the University of Nottingham.

## Ethics Statement

The Heart and Brain Study was reviewed and approved by the University of Oxford Medical Sciences Interdivisional Research Ethics Committee (Reference R57135). The Whitehall II Imaging Sub-Study was granted ethical approval by the University of Oxford Central University/Medical Science Division Interdisciplinary Research Ethics Committee (CUREC/MSD-IDREC), under the specific protocol: “Predicting MRI abnormalities with longitudinal data of the Whitehall II sub-study” (MSDIDREC-C1-2011-71) and the generic “Protocol for non-invasive magnetic resonance investigations in healthy volunteers” (MSD/IDREC/2010/P17.2). The patients/participants provided their written informed consent to participate in this study.

## Author Contributions

SS, CM, DB, SC, and KE: conceptualization. SS, DB, SC, AK, AH, PJ, SR, EZ, LG, TO, MC, MAC, and NB: methodology. DB, SR, TO, MC, MAC, NB, and AK: software. SS, JP, DJ, SG, SR, and DB: investigation. SS, DB, SC, LG, TO, MC, MAC, NB, AK, SG, VS, and MJ: formal analysis. SS, SR, DB, SC, KE, JD, and CM: resources. SS, JP, SG, and KE: data curation. SS, CM, KE, and PJ: funding acquisition. SS, CM, KE, PJ, MK, AS-M, and JD: supervision. SS, CM, and KE: project administration. SS: writing – original draft. All authors contributed to the article and approved the submitted version.

## Conflict of Interest

JD reports provision of medical consulting for the Brain Protection Company Ltd. MC has received royalties for the commercial licensing of FSL and VEASL software tools. TO has received royalties for the commercial licensing of a patent relating to VEASL analysis. The remaining authors declare that the research was conducted in the absence of any commercial or financial relationships that could be construed as a potential conflict of interest.

## Footnotes

1 https://www.oxdare.ox.ac.uk/home

2 https://git.fmrib.ox.ac.uk/falmagro/UK_biobank_pipeline_v_1

3 https://github.com/Washington-University/HCPpipelines/wiki/FAQ

4 http://www.icp-network.org/

5 https://issues.dpuk.org/eugeneduff/wmh_harmonisation/-/tree/master/BIANCA_training_datasets
