# Supplementary material for: Study Protocol: The Heart and Brain Study
Source: Front Physiol. 2021 Mar 31;12:643725. doi: 10.3389/fphys.2021.643725 (PMC8046163; doi:10.3389/fphys.2021.643725)
Supplement: Supplementary file 1 [file Table_1.DOCX]

**Supplementary Materials**

**The Heart-Brain Study Feedback Form for Cerebrovascular Reactivity Scans**

1. Did you notice when you were breathing different gases (please circle one):

YES / NO

2. Did you experience the following? Please rank from 0 (no effect) to 10 (severe effect):

|  | None |  |  |  |  |  |  |  |  | Severe | |
| --- | --- | --- | --- | --- | --- | --- | --- | --- | --- | --- | --- |
| Breathlessness | 0 | 1 | 2 | 3 | 4 | 5 | 6 | 7 | 8 | 9 | 10 |
| Claustrophobia | 0 | 1 | 2 | 3 | 4 | 5 | 6 | 7 | 8 | 9 | 10 |
| Anxiety | 0 | 1 | 2 | 3 | 4 | 5 | 6 | 7 | 8 | 9 | 10 |
| Dizziness | 0 | 1 | 2 | 3 | 4 | 5 | 6 | 7 | 8 | 9 | 10 |

3. What was the most unpleasant part of today’s scan?   ___________________________

4. Did you notice any change in smell and/or taste?    YES / NO

5. Please rate the discomfort you experienced from different aspects of the scan,

    from 0 (no discomfort) to 10 (severe discomfort, wanted to stop the scan):

|  | None |  |  |  |  |  |  |  |  | Severe | |
| --- | --- | --- | --- | --- | --- | --- | --- | --- | --- | --- | --- |
| Being inside the MRI scanner | 0 | 1 | 2 | 3 | 4 | 5 | 6 | 7 | 8 | 9 | 10 |
| Wearing the breathing mask/apparatus | 0 | 1 | 2 | 3 | 4 | 5 | 6 | 7 | 8 | 9 | 10 |
| Breathing different gases | 0 | 1 | 2 | 3 | 4 | 5 | 6 | 7 | 8 | 9 | 10 |

6. Would you be willing to participate in a similar experiment again?  YES / NO / MAYBE

7. Any other comments?
